# Children

**Published:** 1894-12-22

**Authors:** 


### CHILDREN.

**East London Hospital for Children**, Shadwell, E. —An excellent institution, the original home of which in Ratcliff Highway was graphically described by Charles Dickens, whose memorable account will live for generations. Unfortunately, the children admitted are in as dire straits now as then, and funds are sadly needed. Secretary, Mr. Thomas Hayes.

**North-Eastern Hospital for Children**, Hackney Road, Shoreditch (founded 1867).—This hospital seems to have been most unjustly neglected by the charitable public for some time past. During the last three years it has had a deficit of over £1,000 a year, and it is now £4,500 in debt. This state of things is, we believe, partly due to its being hidden away from the gaze of the well-to-do classes in the midst of the poorer districts of London, and partly to the fact that it is too poor to spend the money necessary to bring its work properly before the public, who, however, we are sure, will at once come forward with substantial help when they learn that the hospital has been brought to such straits as to be absolutely without available funds, while over £800 of its accounts for the past three months remain unpaid. The average number of out-patients annually is over 15,000, and of in-patients 690. Secretary, T. Glenton-Kerr; Matron, Miss E. W. Curno; City office, 27, Clement's Lane, E.C; Bankers, Barclay, Bevan, and Co.

**The Hospital for Sick Children**, Great Ormond Street, London, W.C.—Founded in 1852, with ten beds, this was the first hospital solely devoted to the sick children of the poor. There are now nearly 200 beds at Great Ormond Street, besides 52 beds at Highgate. About 1,700 in-patients, besides about 22,000 new out-patients, have been treated in 1894. £1,500 in donations wanted before Christmas to meet ordinary monthly bills. £1,000 asked for in new annual subscriptions to meet cost of maintenance. £4,000 required to free the hospital from the burden of debt on the new building. Secretary, Adrian Hope; Lady Superintendent, Miss Smedley.

**Victoria Hospital for Children**, Queen's Road, Chelsea, and Victoria Convalescent Home, Broadstairs.—Established at Chelsea in 1866 for the relief of the sick and suffering children of the poor, this unendowed hospital seeks further subscriptions to help it to carry on its work. The expenditure, which is only 19s. per patient, yet reaches £7,000 to £8,000 a year, and this has to be raised entirely by voluntary subscriptions. Secretary, Commander W. C. Blount, R.N.; Matron, Miss Hamilton.

